# Associations Between the Length of Time from Transgender Identity Recognition to Hormone Initiation and Smoking Among Transgender Youth and Young Adults

**DOI:** 10.1089/trgh.2018.0002

**Published:** 2018-05-01

**Authors:** David D. Menino, Sabra L. Katz-Wise, Ralph Vetters, Sari L. Reisner

**Affiliations:** ^1^ Division of General Pediatrics, Boston Children's Hospital, Boston, Massachusetts.; ^2^ Division of Adolescent/Young Adult Medicine, Boston Children's Hospital, Boston, Massachusetts.; ^3^ Department of Pediatrics, Harvard Medical School, Boston, Massachusetts.; ^4^ Sidney Borum Jr. Health Center, Fenway Health, Boston, Massachusetts.; ^5^ The Fenway Institute, Fenway Health, Boston, Massachusetts.; ^6^ Department of Epidemiology, Harvard T.H. Chan School of Public Health, Boston, Massachusetts.

**Keywords:** gender affirmation, hormones, smoking, tobacco, transgender

## Abstract

**Purpose:** Gender-affirming medical interventions have been associated with mental health improvements among transgender individuals. However, little is known about optimal timing of these interventions as they relate to stress-sensitive behaviors such as smoking.

**Methods:** We analyzed electronic health record data from transgender youth and young adults to examine whether longer duration from transgender identity recognition to hormone initiation was associated with smoking behaviors.

**Results:** Longer duration from age of identity recognition to age of cross-sex hormone initiation was associated with increased odds of current and lifetime smoking.

**Conclusion:** Future research should investigate other potential risk factors associated with transgender-specific stressors for this population.

## Introduction

Transgender adolescents and young adults are at high risk for a myriad of adverse health outcomes with regard to mental health, HIV infection, and substance use.^1–3^ To date, little data exist on the prevalence of cigarette smoking among transgender youth and young adults. Tobacco is the leading cause of preventable disease and death in the United States, resulting in approximately half a million premature deaths each year.^4^ Despite a decline in current cigarette smoking among adults from 20.9% in 2005 to 15.1% in 2015, it is well documented that individuals who identify as lesbian, gay, or bisexual (LGB) are still disproportionately affected, reporting higher prevalence of smoking than heterosexuals.^4,5^

Similarly, higher trends also exist for transgender than for cisgender (nontransgender) individuals. One national study (*n*=168) reported higher past 30-day use of any cigarette/cigar/e-cigarette product (39.7% vs. 25.1%) and current use of cigarettes (35.5% vs. 20.7%), cigars (26.8% vs. 9.3%), and e-cigarettes (21.3% vs. 5.0%) among transgender adults than among cisgender adults (all *p* values ≤0.003).^6^ In the context of elevated smoking prevalence among transgender versus cisgender people, research is needed to learn about mechanisms that increase risk for smoking in transgender individuals.^6^

Transgender individuals may be at higher risk of developing smoking behaviors earlier than their cisgender peers. Of note, a previous study reports 14.3 years as the average age of smoking initiation in a sample of daily smokers (*n*=167) who identify as transgender adult women; this is younger than the average age reported for cisgender men (15.6 years) and cisgender women (16.7 years) in a nationally representative sample (*n*=16,985).^7,8^

Indeed, transgender identity development may be a period in which transgender-specific minority stressors place transgender adolescents at increased risk for psychiatric morbidity and developing maladaptive coping behaviors such as substance use.^1,3,9–11^ These minority stressors, or stressors that come with membership in a stigmatized group, can be broadly categorized as either “distal” objective stressors (e.g., stigma and discrimination based on transgender identity) or “proximal” subjective stressors (e.g., anticipated stigma or internalized transphobia).^3^

Gender dysphoria (discomfort with the mismatch between one's gender assigned at birth and one's gender identity) is conceptualized as a proximal form of minority stress. Psychiatric morbidity is also significantly associated with smoking among cisgender individuals.^12^ Although general smoking data among transgender adolescents are limited, associations of minority stress and increased smoking behaviors have previously been documented in transgender youth.^3^

Investigating factors that potentially play a role in mitigating transgender-specific stressors may be important for elucidating transgender adolescents' risk for developing smoking behaviors. One such factor may be gender affirmation: the interpersonal, interactive process whereby persons receive social recognition and support for their gender identity and expression.^13^ Previous studies show improved mental health outcomes and reduced gender dysphoria after gender-affirming medical interventions (e.g., cross-sex hormone therapy), with prior work suggesting that psychiatric symptoms may be secondary to incongruity between one's gender identity and gender assigned at birth.^10,14–16^

Although representative statistics regarding the prevalence of undergoing cross-sex hormone therapy among transgender individuals are lacking, cross-sex hormone therapy is recommended by current guidelines to treat individuals experiencing gender dysphoria.^17^ Despite these recommendations, less is known about the optimal timing of initiating cross-sex hormones, particularly in relation to the onset of health risks and other unhealthy coping behaviors such as smoking.

This study sought to formatively investigate the association between the length of time from the age at which individuals recognize their transgender identity (i.e., that they have a gender identity that does not match their assigned gender at birth) to the age at which they initiate cross-sex hormone therapy, and cigarette smoking, an unhealthy stress-sensitive health indicator,^18^ in a community-based clinical sample of transgender youth and young adults who had initiated hormones. A longer duration between these two time points may be conceptualized as a transgender-specific developmental stressor that may result from experiencing gender dysphoria for a longer duration. We hypothesized that longer duration between transgender identity recognition and initiation of cross-sex hormone therapy would be associated with elevated probability of current and lifetime cigarette smoking in this patient population.

## Methods

A retrospective cross-sectional analysis of electronic health record (EHR) data was conducted using records extracted in 2014 from 180 transgender patients seen between 2002 and 2011 at an urban community-based health clinic that serves youth and young adults of ages 12–29 years in Boston, Massachusetts.

A standardized data collection protocol was implemented, whereby a tool was developed and utilized for EHR data extraction to standardize chart review. Variables were extracted from the EHR through a combination of Structured Query Language queries (e.g., demographic and some medical information such as “risk factors” including smoking status) and individual manualized chart review of physician clinical visit narratives (e.g., gender identity development). Analyses were limited to data from patients who reported both their age of transgender identity recognition and age of ever having initiated cross-sex hormone therapy. Data from a total of 46 transgender patients were used for the purpose of this study. All study procedures were IRB approved.

A time difference variable was created by subtracting the reported age of transgender identity recognition from the reported age of hormone initiation. Multivariable logistic regression models adjusted for demographics such as age at first clinical visit, gender identity (FTM: female-to-male, MTF: male-to-female), and race/ethnicity (white non-Hispanic, and racial/ethnic minority). Other covariates were added to adjust for variables that may influence smoking outcomes, such as psychiatric morbidity (having ever received a physician-endorsed diagnosis of depression, anxiety disorders, bipolar disorder, attention deficit hyperactivity disorder (ADHD), etc., per *Diagnostic and Statistical Manual of Mental Disorders*, Fourth Edition, Text Revision [DSM-IV-TR] criteria^19^) and other confounding sources of gender affirmation subsumed under one variable: family support for transgender identity, legal name change, legal gender identification (ID) change, and involvement with transgender organizations.

These models were fit to examine whether the time difference variable was independently associated with an increase in the odds of current and lifetime cigarette smoking. Adjusted odds ratios and 95% confidence intervals were estimated.

## Results

The study sample (*n*=46) had a mean age of 19.2 years (SD=2.96) (Table 1). Seventy-six percent of participants identified as white non-Hispanic and 24% identified as people of color (racial/ethnic minority); 63% identified their gender identity as FTM and 37% as MTF. The average age of transgender identity recognition was 12.13 years (SD=6.15) and the average age of cross-sex hormone initiation was 19.76 years (SD=2.67); and the average time difference between the two aforementioned variables was 7.63 years (SD=5.40).

**Table 1.** **Characteristics of Transgender Youth and Young Adults (*n*=46)**

	Mean (SD)	Range	%	*n*
Health indicators				
Current cigarette smoking	—	—	21.74	10
Lifetime cigarette smoking	—	—	30.43	14
Developmental variables				
Age of hormone initiation (years)	19.76 (2.67)	14.00–26.00	—	—
Age of identity recognition (years)	12.13 (6.15)	4.00–25.00	—	—
Time difference^a^	7.63 (5.40)	0.00–18.00	—	—
Age at first clinical visit (years)	19.20 (2.96)	14.00–29.00	—	—
Gender identity				
FTM	—	—	63.04	29
MTF	—	—	36.96	17
Race/ethnicity				
Racial/ethnic minority	—	—	23.91	11
White non-Hispanic	—	—	76.09	35
Psychiatric morbidity				
Depression	—	—	54.35	25
Generalized anxiety disorder	—	—	36.96	17
Bipolar disorder	—	—	10.87	5
PTSD	—	—	10.87	5
ADHD	—	—	13.04	6
Other^b^	—	—	17.39	8
Gender affirmation				
Family support^c^	—	—	75.00	33
Active in transgender organizations^d^	—	—	48.84	21
Legal gender ID changed^e^	—	—	9.52	4
Legal name changed	—	—	26.09	12

^a^ Age of hormone initiation—age of identity recognition.

^b^ Other diagnoses such as adjustment, eating, developmental, and other anxiety disorders (e.g., obsessive compulsive disorder).

^c^ Family support (*n*=44).

^d^ Active in transgender organizations (*n*=43).

^e^ Legal gender ID changed (*n*=42).

ADHD; attention deficit hyperactivity disorder; FTM, female-to-male; ID, identification; MTF, male-to-female; PTSD, post-traumatic stress disorder.

More than half of the sample had ever received a DSM-IV-TR diagnosis, with diagnoses ranging from depression (54%), generalized anxiety disorder (37%), bipolar disorder (11%), post-traumatic stress disorder (PTSD) (11%), ADHD (13%), and other diagnoses entered into the model together that encompassed adjustment, eating, developmental, or other anxiety disorders, such as obsessive compulsive disorder (17%).

With regard to reported sources of gender affirmation, 75% indicated that the majority of their family members were supportive of their transgender identity, 49% reported active involvement in transgender organizations, 9% had changed their gender marker on their legal IDs (either state ID or driver's license), and 26% had legally changed their names.

Overall, ∼22% of transgender youth and young adults currently smoked and 30% had ever smoked (Table 1). In multivariable models, the time difference variable was found to have statistically significant associations with both current and lifetime cigarette smoking (Table 2). Patients who had a longer duration between the age of transgender identity recognition and the age of cross-sex hormone therapy initiation had a 23% increased odds of current cigarette smoking (*p*<0.026) and a 31% increased odds of lifetime cigarette smoking (*p*<0.007).

**Table 2.** **Multivariable Logistic Regression Models: Smoking Health Indicators (*n*=46)**

	Health indicator 1: current cigarette smoking		Health indicator 2: lifetime cigarette smoking	
	AOR (95% CI)	*p*	AOR (95% CI)	*p*
Independent variable				
Time difference^a^ (adjusted)	1.23 (1.03–1.48)	0.024	1.31 (1.08–1.59)	0.007
Time difference^a^ (unadjusted)	1.20 (1.03–1.39)	0.019	1.22 (1.06–1.40)	0.005
Covariates				
Age in years	0.95 (0.67–1.34)	0.762	0.88 (0.60–1.29)	0.506
FTM vs. MTF	0.15 (0.02–1.16)	0.069	0.17 (0.02–1.30)	0.088
POC^b^ vs. white non-Hispanic	0.11 (0.00–2.07)	0.139	0.04 (0.00–1.03)	0.052
Psychiatric morbidity^c^	0.61 (0.06–6.03)	0.674	0.90 (0.10–8.26)	0.928
Gender affirmation^d^	0.21 (0.02–2.05)	0.180	0.10 (0.01–1.50)	0.096

^a^ Age of hormone initiation—age of identity recognition.

^b^ Person of color; racial/ethnic minority.

^c^ Depression, generalized anxiety disorder, bipolar disorder, PTSD, ADHD, adjustment, eating, developmental, and other anxiety disorders (e.g., obsessive compulsive disorder).

^d^ Family support, active in transgender organizations, legal gender marker on ID changed, and legal name change.

AORs, adjusted odds ratios; CI, confidence interval.

## Discussion

Longer duration of time between age at which individuals recognized their transgender identity and age at which they initiated cross-sex hormone therapy was associated with elevated odds of current and lifetime cigarette smoking among transgender adolescents and young adults in this clinical sample. Although this study did not include measures of gender dysphoria, it is possible that longer duration between identity recognition and hormone initiation may prolong and/or exacerbate this transgender-related stressor,^11^ which may, in turn, lead to maladaptive coping behaviors such as smoking. This is plausible given that the smoking variables were still significantly associated with the time difference variable even after controlling for psychiatric morbidity and other sources of gender affirmation.

Existing recommendations for gender-affirming medical interventions for adolescents promote a staged process of hormone initiation: pubertal blockers (e.g., gonadotropin-releasing hormones) are administered to inhibit development of secondary gender characteristics during puberty, after which puberty suppression may continue for several years before a decision is made to initiate feminizing/masculinizing hormones.^11^ Our data set did not include a measure of whether and how long patients were administered pubertal blockers before cross-sex hormone initiation. However, our preliminary findings, along with documented improvements in mental health, reported reductions in harassment, and improved quality of life in transgender individuals after gender-affirming medical intervention, suggest that earlier timing of cross-sex hormone initiation after gender recognition, while also considering appropriate pubertal timing, may have protective effects for preventing smoking behaviors among transgender adolescents.^10,14–16^

There are limitations to this formative analysis. Smoking data were collected through nonstandardized self-report to a provider; thus, there is some risk that patients may have under-reported smoking behaviors. Future studies should use validated measures to assess smoking behaviors, as well as methods that confirm nicotine content in the body (e.g., cotinine levels).

The small sample size in this study reduces the generalizability of results and power of our analysis. Larger sample sizes should be employed to investigate whether smoking outcomes are consistent with our results, as well as to examine whether there are other nonparenteral substances (e.g., alcohol and marijuana) in addition to smoking that may be significantly associated with the time difference variable. Future studies would also benefit from larger sample sizes that allow for stratification of results by gender identity (i.e., FTM vs. MTF), as existing literature indicates that progesterone and estradiol may influence smoking behavior. Therefore, hormone therapy may have a direct effect on smoking behavior.^20^

Our analyses relied on retrospective cross-sectional data, which did not allow for examination of temporal associations between age and cigarette smoking. Longitudinal research is needed to elucidate cigarette smoking risk among transgender youth and young adults, including the extent to which pubertal blockers and early cross-sex hormone initiation affect smoking behaviors over time for younger transgender adolescents. We also did not have information on whether patients desired to initiate hormone therapy at an earlier time, which may provide additional insight as to whether, and how much, delays in hormone therapy may affect smoking behaviors. Including an additional time difference variable between age at which patients desired hormone therapy and age at which hormone therapy was initiated would be beneficial.

Lastly, this study uniquely focuses on transgender youth and young adults who had sought hormone therapy. Although there are currently no requirements regarding smoking status for medical gender affirmation eligibility, current recommendations state that all transgender women who smoke should be counseled on tobacco risks and cessation options, as feminizing (e.g., estrogen) hormone therapy is associated with an increased risk of venous thromboembolism.^21^ Our data do not contain information regarding tobacco cessation or abstinence after hormone therapy; future studies would benefit by taking these variables into account.

Future research should investigate other gender-affirming medical interventions (e.g., gender-affirming surgeries) and their potential benefits in relation to smoking. In addition, not all individuals who identify as transgender may seek cross-sex hormone therapy or medical gender affirmation, and current standards of care dictate that transgender persons be provided only those interventions they wish to access.^11^ It will be important to conduct research to understand smoking behaviors and related risks in transgender youth and young adults who do not wish to medically affirm their gender.

## Conclusion

Despite limitations, this study contributes new knowledge regarding risk of cigarette smoking in this patient population. Results suggest that longer duration between age of transgender identity recognition and cross-sex hormone initiation may be a transgender-specific developmental stressor associated with increased risk for smoking behaviors among transgender youth and young adults.

## Abbreviations Used

ADHD: attention deficit hyperactivity disorder
DSM-IV-TR: *Diagnostic and Statistical Manual of Mental Disorders*, Fourth Edition, Text Revision
EHR: electronic health record
FTM: female-to-male
MTF: male-to-female
PTSD: post-traumatic stress disorder
